# Determination of nitric oxide metabolites, nitrate and nitrite, in *Anopheles culicifacies *mosquito midgut and haemolymph by anion exchange high-performance liquid chromatography: plausible mechanism of refractoriness

**DOI:** 10.1186/1475-2875-7-71

**Published:** 2008-04-28

**Authors:** Arun Sharma, Kamaraju Raghavendra, Tridibesh Adak, Aditya P Dash

**Affiliations:** 1 Protein Biochemistry Laboratory, National Institute of Malaria Research (ICMR), 22 Sham Nath Marg, Delhi- 110 054, India; 2 Molecular Entomology Laboratory, National Institute of Malaria Research (ICMR) 22 Sham Nath Marg, Delhi- 110 054, India; 3 Entomology Laboratory, National Institute of Malaria Research (ICMR) 2, Nanak Enclave, Delhi-110 009, India; 4 National Institute of Malaria Research (ICMR), 22 Sham Nath Marg, Delhi- 110 054, India

## Abstract

**Background:**

The diverse physiological and pathological role of nitric oxide in innate immune defenses against many intra and extracellular pathogens, have led to the development of various methods for determining nitric oxide (NO) synthesis. NO metabolites, nitrite (NO_2_^-^) and nitrate (NO_3_^-^) are produced by the action of an inducible *Anopheles culicifacies *NO synthase (AcNOS) in mosquito mid-guts and may be central to anti-parasitic arsenal of these mosquitoes.

**Method:**

While exploring a plausible mechanism of refractoriness based on nitric oxide synthase physiology among the sibling species of *An. culicifacies*, a sensitive, specific and cost effective high performance liquid chromatography (HPLC) method was developed, which is not influenced by the presence of biogenic amines, for the determination of NO_2_^- ^and NO_3_^- ^from mosquito mid-guts and haemolymph.

**Results:**

This method is based on extraction, efficiency, assay reproducibility and contaminant minimization. It entails de-proteinization by centrifugal ultra filtration through ultracel 3 K filter and analysis by high performance anion exchange liquid chromatography (Sphereclone, 5 μ SAX column) with UV detection at 214 nm. The lower detection limit of the assay procedure is 50 pmoles in all midgut and haemolymph samples. Retention times for NO_2_^- ^and NO_3_^- ^in standards and in mid-gut samples were 3.42 and 4.53 min. respectively. Assay linearity for standards ranged between 50 n*M *and 1 m*M*. Recoveries of NO_2_^- ^and NO_3_^- ^from spiked samples (1–100 μ*M*) and from the extracted standards (1–100 μ*M*) were calculated to be 100%. Intra-assay and inter assay variations and relative standard deviations (RSDs) for NO_2_^- ^and NO_3_^- ^in spiked and un-spiked midgut samples were 5.7% or less. Increased levels NO_2_^- ^and NO_3_^- ^in midguts and haemolymph of *An. culicifacies *sibling species B in comparison to species A reflect towards a mechanism of refractoriness based on AcNOS physiology.

**Conclusion:**

HPLC is a sensitive and accurate technique for identification and quantifying pmole levels of NO metabolites in mosquito midguts and haemolymph samples that can be useful for clinical investigations of NO biochemistry, physiology and pharmacology in various biological samples.

## Background

Potential control efforts for malaria mainly include vaccine development, vector control and development of new anti-malarial drugs [1]. Vector control has become increasingly difficult due to mosquito resistance to insecticides [2] and efforts to replace natural vectors with mosquitoes that do not support parasite development are under study and may contribute to malaria control in long term [3,4]. Advances in the molecular genetic manipulations of insect species have led to speculation that malaria could be controlled through genetic alterations of *Anopheline *mosquitoes rendered refractory to *Plasmodium *growth and differentiation [5,6]. While assessing natural susceptibility of *Anopheles culicifacies sensu lato *from different geographical areas against *Plasmodium vivax *infection, an iso-female line was found, which was found to be 100% refractory to *Plasmodium vivax *infection. This iso-female line was later identified as *An. culicifacies *species B [7].

The mosquito midgut is the first major site of interaction between the parasite and the mosquito. Failure of the parasite to negotiate this environment can be a barrier for development and is likely the main cause of mosquito refractoriness. The sporogonic development of *Plasmodium*, from gamete to oocyst formation, takes place in the lumen and epithelium of the mosquito midgut [8,9] and some mosquito-specific factors probably determine the outcome of this sporogonic development [10]. It has long been recognized that mosquitoes possess highly effective innate defense mechanisms of both cellular and humoral nature [11,12]. Such responses may be important for the vectorial capacity of the mosquito and understanding of parasite-vector interactions and mechanism of refractoriness. It was also shown by Luckart *et al *[13] that a nitric oxide synthase (NOS) gene in *Anopheles stephensi *is transcriptionally activated at a modest level after malaria infection to limit the development of parasites [13,14]. Induction of *AcNOS (An. culicifacies *NOS) expression is proportional to the intensity of parasite infection and is detectable in the midgut by 6 h post infection [15].

NO metabolites, nitrite (NO_2_^-^) and nitrate (NO_3_^-^) are produced by the action of an inducible *An. culicifacies *NO synthase (AcNOS) in mosquito midguts and may be central to anti-parasitic arsenal of these mosquitoes [15,16]. Measurements of nitrite (NO_2_^-^) and nitrate (NO_3_^-^) in midguts and haemolymph of mosquitoes are proposed indices to reflect accurately cellular nitric oxide production and represents a considerable analytical challenge. The diverse physiological and pathological roles of nitric oxide in innate immune defenses against many intra and extracellular pathogens, have led to the development of various methods for determining nitric oxide (NO) synthesis [16-25]. Because NO is a free radical molecule released by cells in picomolar to nanomolar ranges and has a very short life, a direct measurement of it is difficult [19]. Measurement of nitrite and nitrate, the stable products of NO oxidation, has often been performed to assess NO synthesis in various biological systems [20,25]. A number of ion exchange and reversed phase ion paired HPLC methods have been developed for measuring nitrite and nitrate in biological systems with detection by either UV-VIS absorbance [21] or fluorescence [22] or chemiluminiscence [23] or conductivity [24]. Most HPLC methods require several purification steps to remove interfering substances such as chloride and biogenic amines and therefore, the results may vary considerably [25-27]. Thus, quantification of pico-mole levels of nitrite and nitrate in biological samples is still a challenge.

The aim of the present study was to determine midgut and haemolymph levels of nitrite and nitrate in *An. culicifacies *refractory species B and compares it with sensitive species A over the course of time to evaluate the mechanism of refractoriness and the performance of a novel HPLC method. The HPLC method offers high sensitivity and specificity as well as easy automation for the determination of pico-mole levels of nitrite and nitrate in mosquito midguts and haemolymph. This procedure may also be suitable for routine determination of NO_2_^- ^and NO_3_^- ^in various other biological fluids/samples.

## Methods

### Materials

HPLC-grade water was purchased from Thomas Baker (India) and was used for the preparation of the mobile phase solution. Double distilled and deionised (DD-water) was used for preparing other solutions. Potassium dihydrogen phosphate (KH_2_PO_4_), Potassium phosphate (K_2_HPO_4_), Potassium nitrite (KNO_2_), Potassium nitrate (KNO_3_) was purchased from Sigma (St. Louis, MO, USA). Intermediate and working standard solutions covered the concentration range reported and were prepared by diluting the stock standard solutions with water. All solutions were stored at 4°C.

### Mosquito rearing and blood-feeding

Indoor-resting wild *An. culicifacies s.l*. adult females were collected from human dwellings by hand catch method and transported to laboratory. The iso-female progenies obtained from wild female mosquitoes were held separately in 30 × 30 × 30 cms cloth cages and kept in the insectary maintained at temperature 27 ± 2°C and 75 ± 5% relative humidity (RH) with photoperiod of 14 hr light and 10 hr dark. Adult mosquitoes were offered water soaked raisins and 1% glucose soaked cotton pads as a source of energy. Few adult female mosquitoes from each F_1 _iso-female progeny were identified to sibling species using species-specific diagnostic inversion genotypes as described by Subbarao et al. [28]. At least 50 to 60 iso-female lines from a particular geographical locality showing species-specific diagnostic inversion genotype of species B were pooled together to establish a strain. This iso-female line was later identified as sibling species B and designated as *P. vivax *refractory strain. The susceptibility of all these species B strains was assessed in the laboratory against *P. vivax*, the predominant human malaria parasite species in India.

In each feeding experiment, cohorts of 50 mosquitoes from each of species A and species B strains starved for 12–16 hours were used [29]. About 2–3 ml of *P. vivax *infected blood was drawn from consenting volunteer patient (aged ≥ 16 yrs) having mature *P. vivax *gametocytes density ranging between 0.05 to 0.5% following human use protocol approved by the Human Ethical Committee of the Centre as described by earlier [30]. All *P.vivax *positive patients were treated with 600 mg chloroquine once on day '0' and 15 mg of primaquine for five consecutive days (adult dose) following national drug policy of National Vector Borne Disease Control Programme (NVBDCP), Government of India.

The mosquitoes were fed on *P. vivax*-infected blood through a membrane feeding system, essentially following the method as described by Adak *et al *[30]. In all feeding experiments, laboratory reared *An. stephensi*, which are highly susceptible to *P. vivax *infection, were also fed parallely along with all species A and species B strains on the same blood sample, to assess the comparative infectiousness to mosquitoes. The controls and infected mosquitoes were fed at the same time to minimize the variations in mosquitoes colonies sampled at different times.

After 30 minutes of feeding, unfed and partially fed mosquitoes from each cohort were removed, only fully engorged mosquitoes were kept securely in 30 × 30 × 30 cms cloth cages in the insectary for subsequent examination of sporogonic development. Minimum of 50% of the surviving *An. culicifacies *and *An. stephensi *mosquitoes from each feeding experiment, fed on the same blood isolate were dissected on day '7' and '9–10' and '14–15' in PBSI (phosphate-buffered saline containing 1 mM EDTA and 1 mg/ml Pefabloc^®^). Subsequently, midguts and haemolymph were collected for further analysis.

### Midgut and haemolymph preparation

All dissections were performed on ice, in PBSI. Midguts and haemolymph samples were simultaneously isolated from individually dissected mosquitoes and were pooled (50 samples) from sugar fed (0 day) and uninfected and infected blood fed mosquitoes (7, 9–10, and 14 days). The controls and infected mosquitoes were fed at the same time to minimize the variations in mosquitoes colonies sampled at different times.

Midguts were dissected from sugar-fed female (0-day) mosquitoes. Midguts from blood-fed mosquitoes were opened by a longitudinal incision and haemolymph was directly collected and pooled. Midguts were thoroughly rinsed three times in ice-cold PBSI to remove all traces of peritrophic matrix and gut contents. Haemolymph and dissected mid-guts were stored at -80°C until processing.

### Samples extraction

Mosquito midguts and haemolymph were sonicated and diluted with equal volume of HEPES buffer (10 m*M *HEPES, 140 m*M *NaCl, 2.7 m*M *KCl, 1 m*M *CaCl_2_, 1 m*M *MgCl_2_, 5 m*M *glucose, 1 mg/ml bovine serum albumin, p*H *7.4) and centrifuged at 1,500 g. Supernatants were obtained from washed midgut and haemolymph samples in HEPES buffer for analysis of NO_2_^- ^and NO_3_^- ^and normalized for their protein content between individual groups. Prior to analysis, samples (200 μl) were centrifuged for 30 min through a Microsep (3.5 ml capacity) 3 K micro concentrator filters (Ultracel YM-3, Millipore Co.) at 11,000 g and 3,000 g respectively to remove all the proteins. Filters had been pre-washed with deionized water with centrifugation and disposal of the wastes.

Aliquots of each individually filtered sample extracts were spiked with 25 μM aqueous KNO_2_^-^/KNO_3_^- ^standards as at this concentration the recovery is 100% (Table 1). 20 μl of spiked sample extracts were directly injected onto the chromatograph. Sample concentrations were corrected for silica column NO_2_^-^/NO_3_^- ^contamination by subtracting the values obtained with water which has also been subjected to extraction procedure.

**Table 1:** Recoveries of NO_2_^- ^and NO_3_^- ^in extracted KNO_2_/KNO_3 _standards (Mean ± SD, n = 8)

Concentration	Recovery (%)	
	
	NO_2_^-^	NO_3_^-^
48 nM	90.7 ± 1.9	28.3 ± 3.4
97 nM	94.5 ± 6.2	54.8 ± 4.3
195 nM	94.7 ± 5.9	87.8 ± 5.1
390 nM	93.4 ± 4.0	80.2 ± 1.5
780 nM	93.6 ± 1.9	93.7 ± 4.5
1.56 μM	93.7 ± 2.9	87.9 ± 3.9
3.12 μM	91.8 ± 1.6	95.2 ± 2.9
6.25 μM	98.5 ± 2.4	98.5 ± 3.1
12.5 μM	97.1 ± 1.8	94.8 ± 1.9
25 μM	99 ± 5.4	100.5 ± 3.2
50 μM	100 ± 5.8	97.3 ± 2.9
100 μM	97.1 ± 2.4	99.2 ± 4.9

### Instrument

The chromatograph comprised a Shimadzu Sil-10AD sample injector port with a 100 μl sample loop, a Shimadzu LC-10AT liquid chromatography pump, a UV-VIS detector SPD10 AV and a Shimadzu LC-workstation (all instruments Shimadzu, Tokyo, Japan). The column (250 × 4.6 mm, Sphereclone 5 μ SAX, Phenomenex Co. USA), was an anion exchanger (SEM based on silica). The mobile phase was pumped at a rate of 1.5 ml/min and consisted of 5 m*M *K_2 _HPO_4 _and 25 MM KH_2_PO_4_, *pH *3.0. The effluent was monitored at 214 nm. The injection volume was 20 μl and the column and samples were kept at 35 ± 5°C and 4 ± 5°C, respectively.

### Calculation of sample NO_2_^- ^and NO_3_^- ^concentrations

Sample NO_2_^- ^and NO_3_^- ^concentrations were calculated by reference to KNO_2_^-^/KNO_3_^- ^standard mixtures. Chromatogram peak areas in preference to peak heights were used in these calculations as these were found to yield more reproducible results. These were measured manually with peak "skimming" being employed where necessary. Where appropriate sample concentrations were corrected for background contamination with NO_2_^- ^and NO_3_^- ^in water and buffer solutions.

### Assay validation

Several criteria associated with the assay validation were undertaken according to ICH guidelines [31] so as to define the method's selectivity, linearity, and lower limit of detection, lower limit of quantitation, precision, accuracy and recovery from biological samples.

### Statistical analysis

Data are expressed as mean and standard error of the mean (n = 3) and analysed using 2-way ANOVA (Friedman test). Probability values of *p *< 0.05 were considered statistically significant.

## Results

**Figure 1:**
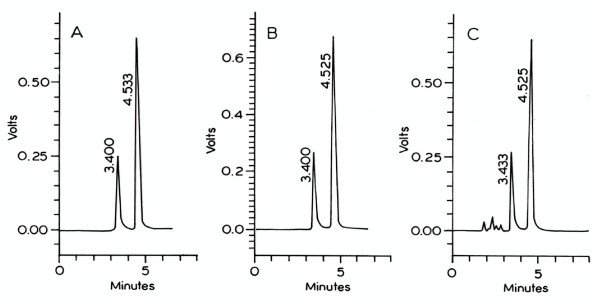
**HPLC analysis of nitrite and nitrate**. Chromatograms of an aqueous standard containing 25 μM nitrite and nitrate (A) and washed and spiked *Anopheles culicifacies *midguts obtained under control conditions (B) and *Anopheles culicifacies *haemolymph.

Typical chromatographic traces of nitrite and nitrate obtained after extraction and direct injection from the standard, mosquito gut samples and mosquito haemolymph samples are presented in Figure 1. The limit of quantification in midguts and haemolymph samples was defined as the lowest concentration of nitrite and nitrate resulting in a signal-to-noise ratio of 3:1. The lowest detection limit was 0.1 μmole/l. The retention times for NO_2_^- ^and NO_3_^- ^in standard solutions were (mean ± SD) 3.400 and 4.533 min. respectively (n = 8).

In mosquito midgut the retention times (n = 8) were 3.400 and 4.525 min. respectively and haemolymph samples the retention times were found to be 3.433 and 4.525 min (Fig. 1). Chromatogram peak identities for samples extracts were confirmed in spiking experiments in which known quantities of NO_2_^- ^and NO_3_^- ^standards were added.

### Recovery

Recovery is expressed as the amount of analyte found as a percentage to the theoretical amount thought to be present in the medium. All samples from mosquitoes were spiked with 1.0, 2.5, 5.0, 10, 25, 50 and 100 μmole/l of each nitrite and nitrate. Ten aliquots of each samples were chromatographed as described in section Chromatography and the resulting peak areas were compared with the peak areas that resulting from the aqueous solutions at the same concentrations (Table 1). The recovery amounts from mosquito midguts were 94.4 ± 4.4, 98.2+6.6, 95.2 ± 2.9, 98.5 ± 3.1, and 99.8 ± 1.9 by coefficients of variations (C.V.) 5.9%, 5.4%, 6.0%, 4.8% and 5.1% for determination of nitrite and nitrate (μmole/l), respectively.

### Intra-day reproducibility

Aliquots of midguts containing 1.0, 2.5, 5.0, 10 25, 50, and 100 μmole/l of nitrite were randomly distributed in different series of assays on the same day (Table 2). The intra-day reproducibility characterized by C.V. was 8.8%, 7.1%, 6.7%, 5.1% and 4.5% for the assays of 1.0, 2.5, 5.0, 10 and 25 μmole/l of nitrite respectively. Similarly, midguts containing 1.0, 2.5, 5.0, 10 and 25 μmole/l of nitrate were randomly distributed in different series of assays on the same day. The intra-day reproducibility characterized by C.V. was 8.7%, 7.2%, 3.9%, 3.1% and 2.2% for the assays of 1.0, 2.5, 5.0, 10 and 25 μmole/l of nitrate, respectively.

**Table 2:** Relative standard deviations (RSDs) and recoveries for NO_2_^- ^and NO_3_^- ^from mosquito midgut spiked with 1–100 μM KNO_2_/KNO_3 _(mean ± SD, n= 6)

Concentration (μM)	NO_2_^-^			NO_3_^-^		
	
	Intra-Assay RSD (%)	Inter-Assay RSD (%)	Recovery (%)	Intra-Assay RSD (%)	Inter-Assay RSD (%)	Recovery (%)
0*	8.3	8.9	-	4.2	5.6	-
1	8.8	9.3	94.4 ± 4.4	8.7	9.9	98.9 ± 2.2
2.5	7.1	9.8	98.2+6.6	7.2	8.5	98.5 ± 2.4
5	6.7	9.9	95.2 ± 2.9	3.9	5.9	97.1 ± 1.8
10	5.1	9.2	98.5 ± 3.1	3.1	2.9	99 ± 5.4
25	4.5	5.3	99.8 ± 1.9	2.2	3.0	100 ± 5.8
50	5.5	4.9	100.5 ± 3.2	2.9	2.6	94.5 ± 6.2
100	3.9	4.7	97.3 ± 2.9	1.2	4.1	94.7 ± 5.9

* Unspiked midgut samples, i.e. midgut samples to which exogenous NO_2_^- ^and NO_3_^- ^has not been added

### Inter-day reproducibility

Aliquots of midguts containing 1.0, 2.5, 5.0, 10, 25, 50, and 100 μmole/l of nitrite respectively were randomly distributed in different series of assays one by one during 10 days using each time the calibration curve of that day (Table 2). The inter-day reproducibility precision values characterized by C.V. was 8.8%, 7.1%, 6.7%, 5.1% and 4.5% for the assays of 1.0, 2.5, 5.0, 10 and 25 μmole/l of nitrite, respectively. Similarly, midguts containing 1.0, 2.5, 5.0, 10 and 25 μmole/l of nitrate were randomly distributed in different series of assays one by one during 10 days using each time the calibration curve of that day. The inter-day reproducibility precision values characterized by C.V. were 9.9%, 8.5%, 7.2%, 2.9% and 3.0% for the assays of 1.0, 2.5, 5.0, 10 and 25 μmole/l of nitrate, respectively.

### Analytical findings

**Figure 2:**
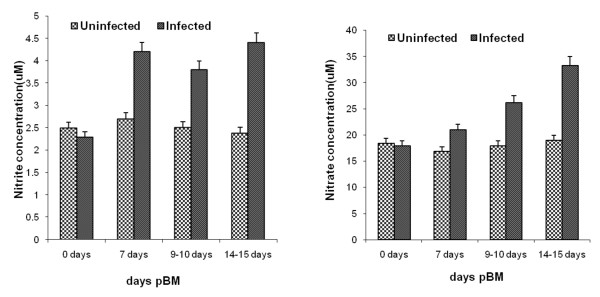
**Haemolymph nitrite/nitrate of blood fed uninfected and blood fed *Plasmodium vivax*-infected *Anopheles culicifacies *species B at 0, 7, 9–10 and 14–15 days pBM using a high performance anion liquid chromatography method**. Means were analysed using a 2-way ANOVA (p < 0.05).

**Figure 3:**
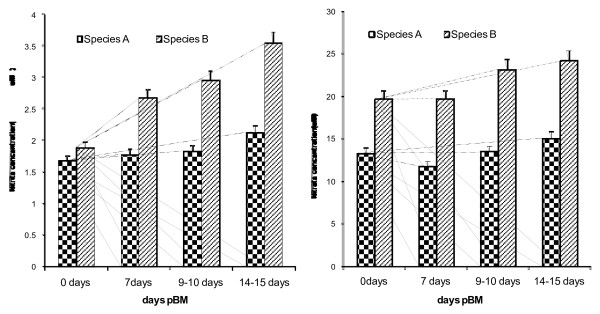
**Midgut nitrite/nitrate of blood fed *Anopheles culicifacies *species A and species B at 0, 7, 9–10 and 14–15 days pBM using a high performance anion liquid chromatography method**. Means were analysed using a 2-way ANOVA (p < 0.05).

Nitric oxide is produced in large quantities during the host defense and immunologic reactions having anti-inflammatory and pro inflammatory properties. Haemolymph and midgut nitrite/nitrate concentrations (Figures 2 and 3) in mosquitoes were found to be significantly higher then the control group (day 0) at any time of study (p < 0.029). There was a significant difference both in *An.culicifaces *species A and Species B nitrite/nitrate levels (Figure 2) at Day 7, Day 9–10, and Days 14–15 (p < 0.04). The higher levels of nitrite/nitrate in midguts of refractory species B following *P. vivax *infected blood feeding provide new insights into the regulation of NO production in refractory species (Figure 3) and may be important for the vectorial capacity of the mosquito to elucidate the mechanism of refractoriness.

## Discussion

Understanding the molecular mechanisms of the innate immune responses of Anopheles against Plasmodium parasites is of great importance for current efforts to develop novel strategies for malaria disease control [32-34]. The parasite undergoes substantial stage-specific losses during its development in the mosquito, which in some cases lead to complete refractoriness of the mosquito against the parasite. The underlying genetics of refractoriness are complex and multi-factorial [4,5]. A variety of factors were shown to negatively affect the development of Plasmodium parasites in the mosquito, in some cases leading to complete transmission blockage [8,32-37]. Malaria parasite infection in anopheline mosquitoes is limited by inflammatory levels of nitric oxide metabolites [13,14,38-41]. As NO is very toxic and highly diffusible, its induction could have deleterious effects on the parasites and ookinetes invading the midgut epithelium [42]. To assess the mechanisms of parasite stasis or toxicity, the biochemistry of these metabolites was evaluated within the blood-filled mosquito midgut as well as in the haemolymph.

Various analytical techniques have been developed to determine nitrite and nitrate, oxidation metabolites of nitric oxide (NO) in biological samples [20,25,27,43]. In previous studies nitrite/nitrate was measured after exposing plasma to copper-cadmium-Zinc catalyst to convert nitrate to nitrite and then adding the effluent of the catalyst product to griess reagent [25]. This procedure carries major disadvantages. Nitrite only is detected by griess reagent while nitrate has to be measured indirectly after reduction to nitrite. It is, therefore, not possible to predict how much of the sample's nitrate is converted to nitrite during the reductive process. Additionally, the complex formation of nitrite and griess reagent is a variable process and may influence the measurement of concentrations [25,44]. Griess reagent reacts with free biogenic amines other then nitrite and may produce false positive results [25]. UV/Vis absorbance [21] and electrochemistry allows simultaneous detection of nitrite and nitrate but is vulnerable to severe interference from chloride present in biological samples [20].

Chemiluminesence [23] and fluorescence detection [22] improve the assay sensitivity and is unaffected by chloride but cannot be applied to simultaneous analysis of nitrite and nitrate. Recently developed fluorometric HPLC method involves the precolumn derivatization of nitrite with 2, 3-diaminonapthalene and enzymatic conversion of nitrite to nitrate [26]. Measurement of nitric oxide itself is complicated by its short half life and would, therefore, require on-line measurement which is suitable for routine use for analysis of biological samples [45].

After an exhaustive investigation, it appears that HPLC, which is a very sensitive, rapid and accurate method with a detection limit for nitrate of 0.1 μM/L, carries none of these disadvantages and the degradation product of nitric oxide, nitrite and nitrate are measured directly. The chromatographic system developed in this study, after investigating alternatives, readily resolved NO_2_^- ^and NO_3_^-^, with peaks being separated by more then a minute as observed by Everett *et al *[45] Anion exchange HPLC coupled with Microsep 3 K filters was found to be suitable and has been advocated by other laboratories as well. Ultrafiltration devices are efficient and permit rapid and easy clean up of the samples whilst avoiding problems relating to contaminants and interfering compounds. However, a Sphereclone column, a phosphate-based eluent and an acidic pH were used to achieve good separation. Particular attention was, however, focused on water quality and washing of plastics and glassware used for the preparation of solutions.

A procedure was developed which may have application in routine extraction and determination of NO_2_^- ^and NO_3_^- ^in biological and clinical samples and offer opportunities for acquisition of data which reflects cellular generation of nitric oxide. The data indicate that circulating levels of nitrite/nitrate, end-products of NO synthesis, were significantly higher in Plasmodium-infected mosquito's midgut. This study implies that the expression of inhibitory mosquito midgut nitric oxide gene elements in response to blood meal may alter the mosquito's vectorial capacity. Mosquito molecules (NO) may be involved in oocyst killing that are refractory to the parasite and may act as an innate immune signal. This may lead to developing novel strategies for controlling the spread of malaria. Detailed knowledge of vector-parasite interactions, particularly in the midgut and the identification of critical mosquito molecules offers prospects for manipulating the vector for the control of malaria.

To date the molecular basis of refractoriness and more generally parasite recognition and killing are not well understood. The identification and cloning of genes conferring mosquito refractoriness to the malaria parasite is critical for understanding malaria transmission mechanisms and holds great promise for developing novel approaches to malaria control. The increase in the production of nitrite/nitrate revealed that NOS may be used as an additional effector gene to block the development of the malaria parasite in mosquitoes [13,14,32]. The present study furthers our understanding of the biochemistry of midgut defense reactions to parasite invasion and how these may influence the efficiency of malaria transmission by anopheline mosquitoes. The present study is highly relevant in view of the current research interest in driving the refractory genes expression into vector populations as a means of interrupting malaria transmission [42] and will provide important insight towards the development of malaria control strategies.

## Conclusion

The procedure described in this paper is suitable for the routine determination of nitrite and nitrate. It has proved a sensitive, accurate and reproducible method. The principal strength of this procedure, is its simplicity. This anion HPLC method coupled with ultrafiltration to reduce protein and salt contaminants has not been used before to measure midgut and haemolymph, nitrite and nitrate concentrations in mosquitoes. This method can be used for the detection, identification and quantitative measurement of all nitric oxide metabolites namely nitrite and nitrate thus making it an effective tool for diagnostic purposes and useful for identifying the AcNOS gene products that may impart refractory phenotype that is associated with the immune response to malaria parasites. Such responses may be important for the vectorial capacity of the mosquito and understanding of parasite-vector interactions and mechanism of refractoriness. This procedure may also be suitable for routine determination of NO_2_^- ^and NO_3_^- ^in various other biological fluids/samples.

## Authors' contributions

AS conceived and supervised the experimental work, carried out mosquito manipulation, HPLC analyses and the writing of the manuscript. KR carried out the mosquito rearing and mosquito dissections. TA carried out the mosquito membrane feeding experiments. APD provided facilities and scientific environment for experimental work. All authors read and approved the final manuscript.
